# Antibacterial Activity of the p53 Tumor Suppressor Protein—How Strong Is the Evidence?

**DOI:** 10.3390/ijms26094416

**Published:** 2025-05-06

**Authors:** Agnieszka Gdowicz-Kłosok, Małgorzata Krześniak, Barbara Łasut-Szyszka, Dorota Butkiewicz, Marek Rusin

**Affiliations:** Center for Translational Research and Molecular Biology of Cancer, Maria Skłodowska-Curie National Research Institute of Oncology, Gliwice Branch, 44-101 Gliwice, Poland; agnieszka.gdowicz-klosok@gliwice.nio.gov.pl (A.G.-K.); malgorzata.krzesniak@gliwice.nio.gov.pl (M.K.); barbara.lasut-szyszka@gliwice.nio.gov.pl (B.Ł.-S.); dorota.butkiewicz@gliwice.nio.gov.pl (D.B.)

**Keywords:** innate immunity, defensin, nutlin-3a, actinomycin D, tuberculosis, MDM2, *Helicobacter pylori*

## Abstract

The p53 tumor suppressor is best known for controlling the cell cycle, apoptosis, DNA repair, and metabolism, but it also regulates immunity and is able to impede the live cycle of viruses. For this reason, these infectious agents encode proteins which inactivate p53. However, what is less known is that p53 can also be inactivated by human pathogenic bacteria. It is probably not due to collateral damage, but specific targeting, because p53 could interfere with their multiplication. The mechanisms of the antibacterial activity of p53 are poorly known. However, they can be inferred from the results of high-throughput studies, which have identified more than a thousand p53-activated genes. As it turns out, many of these genes code proteins which have proven or plausible antibacterial functions like the efficient detection of bacteria by pattern recognition receptors, the induction of pro-inflammatory pyroptosis, the recruitment of immune cells, direct bactericidal activity, and the presentation of bacterial metabolites to lymphocytes. Probably there are more antibacterial, p53-regulated functions which were overlooked because laboratory animals are kept in sterile conditions. In this review, we present the outlines of some intriguing antibacterial mechanisms of p53 which await further exploration. Definitely, this area of research deserves more attention, especially in light of the appearance of antibiotic-resistant bacterial strains.

## 1. Introduction

In contrast to the well-documented association between p53 and viruses, the relationship between bacterial infections and the p53 pathway has not been investigated as thoroughly. After all, p53 was discovered as a cellular molecule binding to a viral protein (reviewed by [1]). Thus, the link between viruses and p53 was known from the very beginning of p53 research, while the connection between bacteria and p53 was noticed much later, initially in the context of infection with cancer-causing *Helicobacter pylori* [2].

In this review, we will show how p53-activated genes could impede the life cycles of bacteria. We will present both well-established facts and less documented models that require further investigation. The role of p53 as an immune regulator was covered by recent reviews [3,4]. In this paper, for each selected gene we will briefly present the strength of the evidence that it is controlled by p53 and what is or might be the mechanism of its antibacterial activity. We were guided in this selection by our transcriptomic analysis, which revealed numerous immunity genes possibly regulated by p53 [5]. First, however, we will summarize the main characteristics of the p53 pathway, which may be relevant to understanding its plausible antibacterial activity.

## 2. The Outline of the p53 Pathway

The comprehensive picture of the functioning of the p53 pathway was presented in recent reviews [6,7,8,9,10]. P53 is a sequence-specific DNA binding protein that acts as a transcriptional regulator of more than 1000 genes, which can be divided into numerous functional groups. Initially, p53 was found to activate genes inhibiting cell cycle progression. Hence, p53 was viewed as a regulator of cell division—the obvious function of a tumor suppressor. However, as more p53-regulated genes were discovered, it became clear that p53 also controls DNA repair and apoptosis. This led to the “guardian of the genome” model of p53 function [11]. As subsequent genes regulated by p53 were identified, the known physiological functions of p53 extended to include metabolism, cellular senescence, autophagy, ferroptosis, translation control, angiogenesis, and self-regulation. It was later realized that p53 plays an important role in immunity, especially in antiviral defense (recently reviewed [12]). Therefore, given its ability to regulate numerous processes, p53 is often referred to as the “guardian of homeostasis”. However, although it has been realized that p53 regulates various cellular functions, it is still viewed in an oversimplified way as the regulator of the cell cycle, DNA repair, and apoptosis.

P53 is an inherently unstable protein that is in a feedback loop with its main negative regulator—MDM2—a ubiquitin ligase, which marks p53 for degradation. Therefore, the steady-state level of p53 in physiological conditions is very low. Nevertheless, when a cell needs to activate p53 its interaction with MDM2 is prevented by various mechanisms, e.g., post-translational modifications of p53 (mostly phosphorylation of its amino-terminus), sequestration of MDM2, or both. This leads to the stabilization of p53, which, as a tetramer, starts to bind to gene promoters and/or enhancers and begins to up-regulate its target genes. P53 is post-translationally modified on many of its amino acid residues, which not only stabilize it but also help to bind it to the transcriptional machinery of the cells, enabling gene activation. The interaction of p53 with MDM2 can be prevented by various small molecules, e.g., nutlin-3a. This leads to the activation of p53 with all of its consequences [13]. P53 also represses many genes, mostly those that drive DNA replication and cell division. Gene silencing occurs predominantly indirectly by promoting the activity of gene repressors [14]. Some genes are activated by p53 indirectly, because p53 promotes the expression of gene regulatory proteins, which, in turn, regulate their own gene targets [15]. Yet, these genes are under the control of p53. Indirectly regulated genes do not have p53 binding sites within their promoters and enhancers. The lack of p53 binding in the vicinity of a gene does not, however, preclude its direct regulation by p53, because some genes are controlled by distant enhancers whose association with the regulated gene is hardly recognized.

The gene regulatory circuits found in bacteria frequently act as on/off switches. Many transcriptional regulators also function in this way in mammalian cells, initiating specific differentiation programs. However, the activity of p53 as a transcriptional regulator can be modulated. Obviously, the biological outcome of p53 activation is often of the “all or nothing” type, e.g., a cell either divides or arrests its cell cycle, or a cell stays viable or undergoes apoptosis. According to a model presented many years ago, the degree of p53 activation can change due to the intensity or combination of stress factors that stimulate p53 [16]. In this model, the level of activation is governed both by the nature and number of post-translational modifications of p53 and by its interactions with other proteins, for example, phosphorylation of serine 46 is a “marker” of strong p53 activation [17,18]. When the activation level of p53 is low, it is phosphorylated on selected N-terminal amino acids and activates the expression of genes that arrest the cell cycle. When the activation level of p53 is high, it becomes additionally acetylated near the C-terminus and promotes the expression of pro-apoptotic genes. This has important physiological significance because low stress usually does not damage cells beyond repair, whereas extensive stress does, and damaged cells must be eliminated [16]. Of course, this is a simplified model, but it illustrates a general pattern. Thus, the outcome of p53 activation depends on the type of stress, stress intensity, and the type of cell. For instance, in some cells strong activation of p53 leads to permanent cell cycle arrest, called cellular senescence, while in other cell types it leads to apoptosis (reviewed by [19]). Sometimes, p53 activation leads to molecular changes that prime a cell for a certain outcome when a given trigger appears. For example, when p53 is strongly activated by simultaneous exposure of A549 cells to two activators of p53, actinomycin D and nutlin-3a (A+N), the cells undergo cell cycle arrest, but, surprisingly, they do not die by apoptosis even though there are many p53 molecules with phosphorylated serine 46. However, when treated cells are additionally exposed to low concentrations of a molecule known as an FAS ligand more than 99% of cells die within 5 h, which does not happen to cells treated with the ligand alone. The sensitivity to the FAS ligand is also increased in cells exposed to actinomycin D or nutlin-3a, but the death rate after this mono-treatment is only 35% [20]. Because apoptosis is an “all-or-nothing” event, stronger activation of p53 by co-treatment with actinomycin D and nutlin-3a resulted in more cells in the population reaching the threshold of sensitivity to the FAS ligand. However, when the result of p53 activation is not all-or-nothing, such as in the case of the glucose metabolism rate or the rate of protein translation [21], then the intensity of the outcome may parallel the degree of p53 activation.

This strength of p53 activation impacts the number of up-regulated genes, which is well-illustrated by experiments involving the simultaneous treatment of cancer cells with two different p53 activators such as the aforementioned actinomycin D and nutlin-3a. In the A549 lung cancer cell line exposed to nutlin-3a, a classic p53 activator that is an antagonist of the negative regulator of p53, MDM2 protein, the treatment induces a strong (at least fourfold) up-regulation of 94 genes. In cells exposed to actinomycin D, which activates p53 by a different mechanism than nutlin-3a, treatment strongly activates 133 genes, while combined treatment (A+N) significantly up-regulates 587 genes. This clearly indicates the synergy between actinomycin D and nutlin-3a in gene activation and is associated with the number of p53 molecules with phosphorylated serine 46. The synergistic increase in the number of up-regulated genes results from the fact that many genes are activated only by the drugs acting together. This allowed for the identification of many new p53 target genes [5]. Interestingly, to the best of our knowledge, the strong activation of p53 by the combination of actinomycin D and nutlin-3a does not stem from extensive cell damage but from the apparent synergy between two p53 activation mechanisms. In cells of various origins with wild-type p53 (e.g., in lung cancer, osteosarcoma, or melanoma cells), we can observe some universal effects of strong p53 activation. Of course, there is an important question about the biological significance of this activation by an exotic combination of substances, but it is plausible that the two mechanisms can be simultaneously activated by some natural stress conditions.

P53-regulated genes can be divided into the so-called core transcriptional program, which comprises approximately 100 genes activated in most cell types by most activators, and into the group of genes activated in a cell- or stress-specific manner [22]. Furthermore, when pluripotent cells start to differentiate, p53 can no longer activate some genes, or genes are up-regulated by other transcriptional regulators and activated p53 can no longer make a difference [23]. A census of p53 target genes found in gene-focused studies yielded the number 346 [24]. This analysis was extended by creating a very useful tool for the identification of plausible p53 target genes [25]. The authors built a web atlas, TargetGeneReg 2.0, that combines information from dozens of projects aimed at the identification of target genes in different cell types (mostly cancer cells) and treatment conditions. Moreover, the tool gives information on p53 binding to gene promoters and enhancers. Based on this information, one can conclude whether a gene is a direct p53 target and how strong the evidence for this is. The tool provides a “p53 expression score”, which reflects the number of studies showing that a gene is activated by p53. The score ranges from −55 to 57. For example, for the archetypal p53-activated gene, *CDKN1A* coding for p21 protein, which is the main cell cycle inhibitor, the score is 57. According to this analysis, the number of genes with a high p53 expression score is 3456, which is disturbingly large because it suggests that a substantial part of the human genome is under the control of p53. However, many of these genes are only marginally activated, for example, twofold. Therefore, when analyzing gene expression, it is important to consider not only whether it is activated in a p53-dependent fashion but also how strong the activation is. The p53 target genes that make the difference in cellular physiology are the ones that show a relatively high-fold change.

When considering the number of genes, it is important to keep in mind that the activation of a given gene by p53 is also governed by the cooperativity of binding between p53 molecules forming a functional tetramer. One of the best-known examples of positive cooperativity is oxygen binding by hemoglobin, which is also a tetrameric protein. The cooperativity in p53 is regulated in part by post-translational modifications of its monomers. Under conditions that do not promote cooperativity, the active p53 tetramer recognizes DNA sites with a very good match to the consensus sequence consisting of two direct repeats of RRRCWWGYYY (R stands for A or G, W stands for A or T, Y stands for C or T). These sites are enriched in cell cycle control genes. In conditions promoting cooperativity, the p53 tetramer additionally binds to sequences that deviate from the consensus. These sequences are enriched in pro-apoptotic genes [6]. Thus, it can be hypothesized that a stress factor that activates a large number of genes induces p53 post-translational modifications, which promote cooperativity. It is also possible that the stress factor promotes post-translational modification of p53, which enables its interaction with more transcriptional co-activators and switching on of additional genes.

The gene regulatory activity of p53 involves acting as a sequence-specific DNA-binding protein, although p53 can also regulate some cellular tasks more directly without binding to DNA. The best-known transcription-independent function is the activation of apoptosis by direct binding to the outer mitochondrial membrane and the promotion of the release of pro-apoptotic cytochrome c into the cytosol (reviewed by [26]). However, p53 was also found to directly inhibit the glucose-6-phosphate dehydrogenase, which catalyzes the first and rate-limiting step of the pentose phosphate pathway. This pathway uses glucose to generate NADPH, which is essential for biosynthetic reactions and antioxidative defense [27].

Since p53 activity is triggered by diverse stress factors, the question arises as to whether p53 is activated by bacterial infections. Bacteria that grow inside cells or in the extracellular space generate various stressors, e.g., foreign metabolites and toxins. The aforementioned actinomycin D, which activates p53, is a natural bacterial product ([28] and references therein). Moreover, the immune system itself, in an attempt to kill bacteria, generates genotoxic stress, e.g., by an oxidative burst of neutrophils [29]. Can p53 be activated by these factors and does it activate genes that slow down the growth of bacteria or kill them? On the other hand, if p53 is an antibacterial protein, bacteria could be expected to make molecules that inactivate p53, like many viruses do. In the next section, we will try to answer these questions.

## 3. The Current Picture of Interactions Between Bacterial Infections and the p53 Pathway

**Figure 1:**
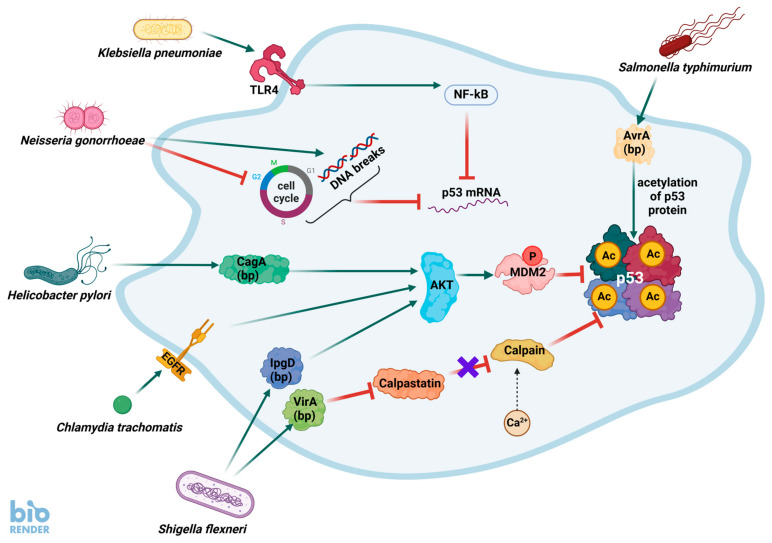
Bacteria antagonize p53 protein by mechanisms of its destabilization or by reducing expression of its mRNA. Bacteria produce proteins (bps), which interact with cellular molecules interfering with the p53 pathway. For instance, one of the proteins (IpgD) produced by *S. flexneri* promotes activation of AKT kinase, which phosphorylates and activates MDM2, which is a major negative regulator of p53 inducing its degradation. Another *S. flexneri* protein (VirA) induces proteolysis of calpastatin, which is a negative regulator of calpain. Liberated calpain snips off the N-terminus of p53. Other bacteria also act through the activation of AKT. For instance, in *H. pylori*, this function is played by the CagA protein. On the other hand, *C. trachomatis* activates EGFR (epidermal growth factor receptor), which by its natural signaling pathway stimulates AKT. *N. gonorrhoeae* arrests the cell cycle and induces DNA breaks in host cells, which in normal conditions would activate p53, but infection with this bacterium, by an unknown mechanism, reduces expression of p53 mRNA. *K. pneumoniae* activates Toll-like receptor 4 (TLR4), which indirectly activates the NF-κB pathway, which through mediators causes the destabilization of p53 mRNA. The only known bacterium that apparently activates p53 is *Salmonella Typhimurium*, which through its AvrA protein promotes p53 acetylation, which is associated with its stabilization and activation.

It is well-established that during infection bacteria produce proteins that directly impact the functioning of the p53 pathway, mostly negatively (Figure 1). Many studies on the relationship between bacteria and p53 focus on *Helicobacter pylori*, which is a bacterial pathogen best studied in the context of carcinogenesis. It is hypothesized that p53 inactivation is a part of the pro-carcinogenic activity of this bacterium. This topic is extensively covered in reviews published 10 years ago [30,31]. These reviews concentrated mainly on the mechanisms employed by bacteria to inactivate p53 and less on the mechanisms used by this protein to counteract or prevent infections. Since then, many new p53-regulated genes have been discovered, mainly during high-throughput studies (reviewed by [25]). This new information can expand our understanding of how p53 may hinder the proliferation and pathogenicity of bacteria. However, many hypotheses generated by the identification of novel p53 target genes await rigorous testing.

Bacterial infections can generate DNA damage, e.g., double-strand breaks [32,33], but these infection-induced DNA alterations rarely lead to p53 activation [33], suggesting that bacteria may somehow actively prevent its stimulation. Activated p53 can apparently interfere with the life cycle of bacteria and, therefore, bacteria have evolved various mechanisms to negatively regulate p53. Intracellular bacteria exploit the resources of their host, for example, by redirecting metabolic pathways to generate nutrients. To hinder bacterial replication, p53 can modulate cellular metabolism. This has been best studied in *Chlamydia trachomatis* infections. This bacterium consumes a large amount of cellular NADPH, which is primarily generated by the oxidative phase of the pentose phosphate pathway. P53 directly inhibits glucose-6-phosphate dehydrogenase, which is the first and rate-limiting enzyme of this pathway responsible for generating reduced NADPH [27]. Lower levels of NADPH can inhibit the growth of *C. trachomatis*, and probably for this reason the pathogen promotes p53 degradation by increasing the activity of MDM2. The destabilization of p53 by MDM2 activation was also discovered during infections with other bacteria such as *Shigella flexneri* and *H. pylori* (reviewed by [30]). In a study published more recently, lipopolysaccharides from *Klebsiella pneumoniae* and other Enterobacteria were found to strongly inhibit the host p53 tumor suppressor pathway via a novel mechanism, namely by destabilizing *TP53* mRNA through a TLR4-NF-κB-mediated inhibition of the RNA-binding factor Wig-1 [34]. Down-regulation of p53 was also found when oral cancer cells were co-incubated with *Enterococcus faecalis* [35].

According to the model proposed 10 years ago, bacteria render p53 inactive because it promotes the formation of metabolic environments that hinder their growth. Unexpectedly, infection with *Salmonella Typhimurium* has the opposite effect on p53, namely causing its stabilization which is associated with acetylation, which induces, as expected, cell cycle arrest. This stabilization of p53 is mediated by the bacterial AvrA protein. It is not known why *S. Typhimurium* infection activates the p53 pathway. Does the bacterium exploit activated p53 to its advantage, e.g., by modulating the inflammatory response [36]? Even though the work was published 15 years ago, there are no answers to these questions. Definitely, pharmacological activation of p53 by nutlin-3a leads to a severe growth defect and complete loss of *C. trachomatis* infectivity [37]. The question is whether p53 does this through metabolic reprogramming, which causes bacteria starvation, or via some other mechanisms. In line with this observation, nutlin-3a restores the p53 activity which is inhibited by *H. pylori* and decreases the survival of infected cells [38]. Therefore, when a bacterium cannot block p53, this protein kills the host cell. Activation of p53 by cisplatin can inhibit mycobacteria proliferation in macrophages [39].

Animal experiments indicate that p53 is generally an anti-inflammatory protein. For example, the lungs of naive p53 knockout mice show genome-wide induction of NF-κB response element-enriched pro-inflammatory genes [40]. Similar conclusions were drawn from experiments with p53 knockout mice infected with *Helicobacter pylori*. Infected animals exhibited increased scores of chronic and active inflammation compared to uninfected controls [2]. This may be due in part to the general antagonism between p53 and the pro-inflammatory NF-κB signaling pathway [41]. This enhanced inflammatory response in p53-deficient mice is associated with increased bacteria clearance after intrapulmonary infection. Despite this, infected p53 knockout mice suffer increased mortality associated with aggravated lung injury, likely caused by the overreacting immune system [40]. This experiment suggests that p53 is an anti-inflammatory protein, and when it is missing the hyperactive immune system becomes more effective at killing bacteria. However, as a collateral effect, it also damages the lungs, leading to the death of animals. This is reminiscent of a cytokine storm that kills a fraction of human patients after bacterial or viral infections [42].

Other researchers infected p53 knockout mice with *Listeria monocytogenes*, a Gram-positive bacterium that often causes invasive diseases in humans and animals, especially infections of the central nervous system. P53 knockout mice were more susceptible to *L. monocytogenes* infection, manifested by a shorter survival time and a lower survival rate. The knockout animals had problems with the eradication of bacteria and exhibited severe clinical symptoms and organ injury, presumably due to abnormal production of some pro-inflammatory cytokines. However, p53-deficient animals showed lower production of interferon-γ (IFN-γ) and guanylate-binding protein (GBP1), coded by the IFN-γ-activated gene [43]. GBP1 is an important element of innate immunity, helping to detect intracellular bacteria by inflammasomes (see below), whereas IFN-γ activates macrophages. Thus, in this model, p53 helps to both eliminate bacteria and survive the infection.

Lim et al. [44] made very interesting observations on the relationship between infection with *Mycobacterium tuberculosis* and p53 activation. Infection with this bacterium is one of the most important health problems worldwide, therefore, it is crucial to understand how this infection affects the p53 pathway and how activated p53 can modify the course of infection. In their experiment, the authors infected murine bone marrow-derived macrophages with a virulent (H37Rv) and attenuated (H37Ra) strain of *M. tuberculosis*. The production of p53 protein was significantly enhanced with infection. Macrophages infected with the attenuated strain produced higher levels of p53 compared to macrophages infected with the virulent strain. Thus, the attenuated strain of *M. tuberculosis* induces a strong accumulation of p53 in murine macrophages. The intracellular survival of *M. tuberculosis* was improved in p53-deleted macrophages compared to wild-type p53 macrophages. In general, induction of p53-dependent apoptosis forced a reduction in the intracellular growth of *M. tuberculosis* in macrophages. Furthermore, nutlin-3 effectively reduced the intracellular survival of mycobacteria in both tuberculosis patients and healthy controls after infection with the attenuated strain. In this model, p53 clearly shows its antibacterial activity, which prompted the authors to suggest that p53 may be a new therapeutic target for tuberculosis therapy [44].

Thus, in many model systems, p53 activation can inhibit the growth of bacteria. However, the details of the antibacterial activity of p53 are poorly studied. In the following sections, we will present how p53 can counteract the bacterial life cycle by the up-regulation of various genes. In this review, we focus on p53-regulated genes encoding proteins helping to fight bacterial infections. Generally we do not discuss antiviral genes. If we do, like in the case of *NLRP1* or *TLR3* genes, it is because they also show some antibacterial functions, which we present below.

## 4. The p53 and Inflammasomes

**Figure 2:**
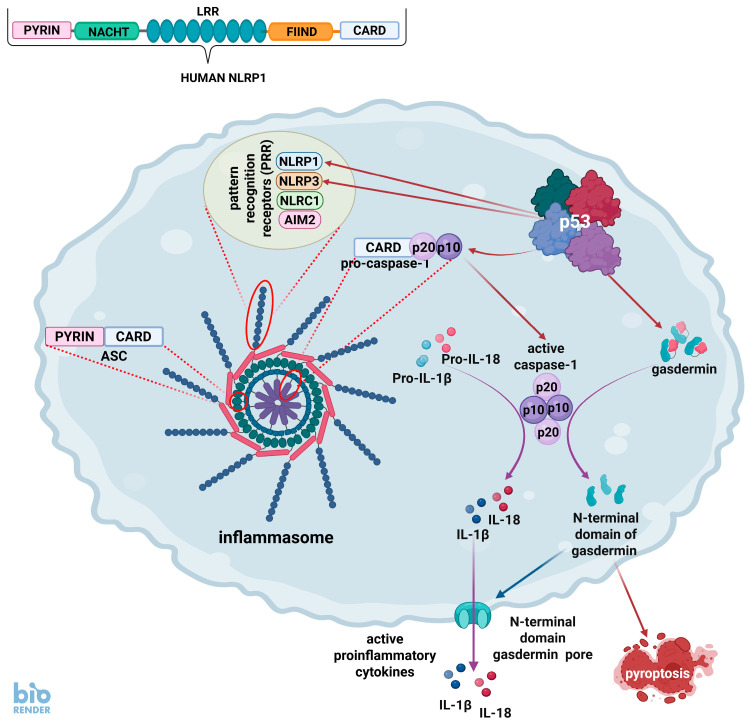
The p53 activates at least three genes, which encode the components of inflammasomes. A simplified model of pro-inflammatory caspase-1 activation. Inflammasomes are multiprotein complexes promoting inflammation. They consist of pro-caspase-1 (localized centrally) linked to various pattern recognition receptors (PRRs, localized peripherally) by the ASC protein. Pro-caspase-1 and ASC are linked through CARD domains, whereas ASC and PRRs are linked through PYRIN domains. Binding of a ligand (e.g., a bacterial molecule) to a PRR induces a series of molecular alterations, which lead to the cleavage of pro-caspase-1 into p10 and p20 domains, which as a dimer of dimers form active-caspase-1. This enzyme cleaves inactive pro-inflammatory IL-1β and IL-18 into active molecules. Moreover, caspase-1 cuts gasdermin proteins, whose N-terminal fragments form pores within the plasma membrane. These pores are used by active cytokines to escape into extracellular space. Moreover, the pores contribute to cell swelling and death, which is called pyroptosis. Interestingly, NLRP1 can directly interact with pro-caspase-1 through the CARD domains of both proteins. The NLRP1 inflammasome is poorly studied, but it can be the predominant inflammasome in human barrier cells. The genes coding these components of the inflammasomes (pro-caspase-1, NLRP1, and NLRP3) are directly activated by p53. Moreover, p53 activates a gene for one of the gasdermins—GSDME.

One of the most important detectors of bacterial infections is the multiprotein complexes called inflammasomes (Figure 2). These structures were discovered in 2002 and described as molecular platforms that trigger the activation of pro-inflammatory caspases, e.g., caspase-1 [45]. More than 20 years of research have revealed the diversity of inflammasomes. They consist of three common constituents that determine their identity. The first component is a pattern recognition receptor (PRR), which recognizes foreign, e.g., bacterial, or damaged molecules. The second element of inflammasomes is an adaptor protein named ASC (apoptosis-associated speck-like protein containing a CARD), which functions as a link connecting the PRR with the third element, which is one of the pro-inflammatory caspases. The inflammasome caspases are different from pro-apoptotic caspases. The major function of the inflammasome is to activate caspases in response to foreign molecules detected by the PRRs. Active caspase cleaves and activates pro-inflammatory interleukins (IL-1β and IL-18) and promotes their release into the extracellular space. Furthermore, caspase cleaves and activates proteins called gasdermins, which form pores in the plasma membrane and trigger pro-inflammatory cell death called pyroptosis. To offer a simplified understanding of pyroptosis, its induction can be divided into several steps. Step 1—binding of foreign molecules to pattern recognition receptors of inflammasomes. Step 2—cleavage of inactive pro-caspase-1 into active molecules consisting of two p10 and two p20 fragments. Step 3—cleavage by caspase-1 of pro-interleukin-1β and pro-interleukin-18 into active molecules. Step 4—cleavage by caspase-1 of gasdermins into active N-terminal fragments. Step 5—formation of plasma membrane pores by N-terminal fragments of gasdermins. Step 6—the escape of active interleukins into extracellular space. Step 7—cell swelling and death. For the sake of clarity we ignored some important processes taking place during pyroptosis, e.g., changes in cellular potassium concentration.

Inflammasomes are generally classified according to the PRR, which they contain, such as NLRP1, NLRP3, NLRC1, and AIM2. Each inflammasome contains only one type of PRR. These receptors activate caspase-1 and form so-called classic inflammasomes. Non-classic inflammasomes contain caspase-4 and caspase-5 and are poorly explored. The most thoroughly studied inflammasome contains NLRP3 and is widely present in immune cells, including granulocytes, dendritic cells, and macrophages, as well as epithelial cells and osteoblasts. It recognizes diverse stimuli, including bacteria, fungi, and viruses. Interestingly, Gong et al. [46] demonstrated that p53 can activate *Nlrp3* gene in the murine macrophage cell line RAW264.7. The p53 expression score of the human *NLRP3* gene is low (six) (Table 1). Our transcriptomic experiments detected *NLRP3* activation only in U-2 OS cell line exposed to actinomycin D and nutlin-3a, which is consistent with the database presented by Fischer et al. [25] showing the activation of *NLRP3* in U-2 OS cells exposed to nutlin-3a and in the NCI-H1299 p53-null lung cancer cell line with ectopically expressed p53. Apparently, the activation of *NLRP3* by p53 is limited only to some cell types or stress conditions.

Another PRR named NLRP1 is unique because it can directly bind to pro-caspase-1, so even though the ASC protein promotes caspase-1 activation in response to NLRP1, it is not absolutely required. This inflammasome recognizes bacterial products, including lethal anthrax toxin, but also SARS-CoV-2, which cleverly fights back and dampens inflammasome activation (reviewed in [53]).

**Figure 3:**
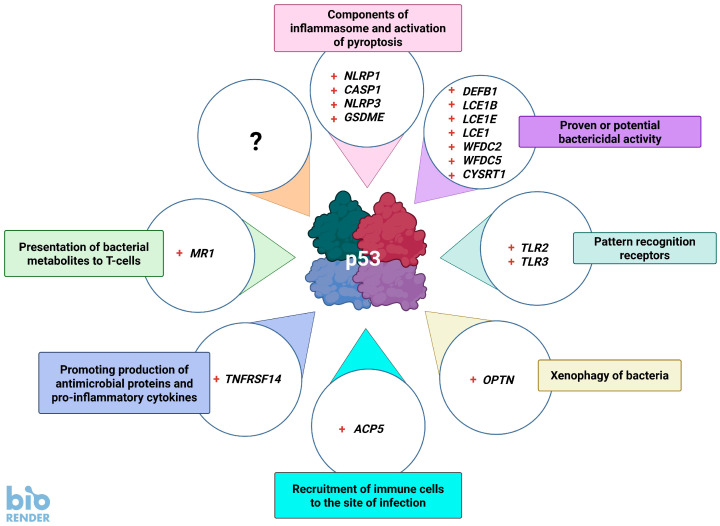
Multiple roles of p53 in antibacterial defense. The genes regulated by p53 involved in the indicated functions and described in this review are shown in circles. The “+” sign means that these genes are up-regulated in a p53-dependent fashion. There are probably more antibacterial genes regulated by p53 that have not been identified yet, hence the question mark.

The *NLRP1* (NLR family pyrin domain containing 1) gene is markedly activated upon exposure of cells to p53-activating agents such as camptothecin, actinomycin D, and nutlin-3a. The latter two compounds strongly synergize in *NLRP1* activation, which is significantly attenuated in p53-deficient cells. The cloned *NLRP1* promoter is robustly activated by ectopically expressed p53 [47]. Transcriptomic data [5] confirm these results and also show that this gene is strongly activated by A+N in the NCI-H460 lung cancer and in the U-2 OS osteosarcoma cell lines (Table 1). It is important to mention that in both examined lung cancer cell lines, NCI-H460 and A549, *NLRP1* is not expressed in cells growing in control conditions [5]. *NLRP1* has a high p53 expression score, and both its promoter and enhancer are bound by p53 [25], which indicates that *NLRP1* is directly activated by p53. Therefore, p53, by controlling this gene, promotes the formation of inflammasomes which can be activated by molecules binding to the NLRP1 receptor (Figure 2 and Figure 3). The NLRP1 inflammasome was first described in human microglia and neuronal cells before being characterized in mice (reviewed by Xu et al. [54]). Interestingly, it is also constitutively expressed in primary keratinocytes, but not in immortalized keratinocyte cell lines [55]. Mice are not a good model for studying plausible human NLRP1 functions because the activation triggers and mechanisms of human NLRP1 and its mouse counterpart are highly divergent. The physiological activators of human NLRP1 have not been identified for a long time. Recently discovered activators are proteins coded by human rhinovirus (HRV) and the aforementioned coronavirus. The long, double-stranded RNA molecules generated during Semliki Forest virus replication can also be detected by NLRP1 (reviewed by [54]). NLRP1 can probably detect the presence of other viruses, whose life cycle involves the formation of long, double-stranded RNA molecules. These data suggest that human NLRP1 primarily recognizes viral infections. The aforementioned anthrax toxin activates the murine counterpart of NLRP1, and data on human NLRP1 activation by the anthrax toxin are sparse (reviewed in [54]). However, two recently published companion studies demonstrated that the human NLRP1 inflammasome can be activated by toxins produced by *Pseudomonas aeruginosa* and *Corynebacterium diphtheriae* [56,57]. Therefore, all these data suggest that human NLRP1 is a versatile sensor of infection with both viruses and bacteria, controlled by p53.

NLRP1 is an element of one type of classic inflammasomes, all of which contain caspase-1. The activation of caspase-1 gene (*CASP1*) by p53 was identified in 2001 before inflammasomes were even discovered [48]. However, at this time it was already known that caspase-1 activates interleukin-1β protein and promotes the secretion of this cytokine. In fact, one of the names of caspase-1 is “IL-1 beta-converting enzyme”. The activation of *CASP1* by p53 was also confirmed by Schlereth et al. [49], who additionally showed that the binding of p53 to the *CASP1* promoter requires strong cooperative binding between p53 monomers. Thus, the binding of p53 to the *CASP1* gene is mechanistically different from the binding of other gene promoters and probably requires a special set of post-translational modifications which promote the cooperative binding of p53 monomers. Interestingly, our observations indicate a very strong synergy between actinomycin D and nutlin-3a in the activation of the *CASP1* gene and confirmed that the activation of this gene is dependent on p53 [47]. The transcriptomic data show very strong activation of *CASP1* in response to A+N in the A549, NCI-H460, U-2 OS, and A375 cell lines (Table 1). All these cells produce virtually no *CASP1* mRNA when growing in control conditions. Hence, the fold change after exposure to A+N is very high because the expression starts virtually from zero in control cells [5]. Other high-throughput experiments also show *CASP1* regulation by p53, although the p53 expression score is moderate, suggesting that this gene is activated by p53 in a subset of cells and/or stress conditions [25]. Its promoter is bound by p53, which indicates direct regulation by this protein [25]. Considering that caspase-1 is an element of inflammasomes recognizing various bacteria, it can be concluded that a very important part of the antibacterial activity of p53 is mediated by *CASP1* activation.

Caspase-1 activated within inflammasomes not only activates interleukins-1β and 18 but also cleaves and activates the proteins named gasdermins that form the pores within the plasma membrane which serve as exit points for activated interleukins and cause cell permeabilization leading to swelling and death, which is called pyroptosis. The gene encoding one of the gasdermins, gasdermin D (*GSDMD*), is up-regulated by A+N co-treatment in A549 cells [5] but has a low p53 expression score (−1). Therefore, there is no convincing evidence that this gene is commonly controlled by p53. However, a related gene, *GSDME* also known as *DFNA5*, encoding another gasdermin was found almost 20 years ago to be directly activated by p53 [50]. According to Fisher et al. [25], it has a high p53 expression score (31) and our transcriptomic experiment demonstrated its up-regulation in the NCI-H460 and A375 cell lines [5]. Thus, p53 can up-regulate at least one gene encoding the pore-forming elements executing pyroptosis.

There is another element of the inflammasome, the IFI16 protein, which is coded by a long-known p53 target gene [58]. This gene is strongly up-regulated by A+N in a p53-dependent manner in the A549 cell line [47]. Interestingly, it has a low p53 expression score (−2) mainly due to the fact that 10 high-throughput studies show that it is negatively regulated by p53 and only 8 studies indicate that p53 activates this gene. There are p53 binding sites within its promoter and enhancer [25]. Transcriptomic analysis of cells exposed to A+N shows that the *IFI16* gene is up-regulated in the A549, NCI-H460, U-2 OS, and A375 cell lines; therefore, it appears that the regulation of IFI16 by p53 is specific to stress or cell type. For example, IFI16 is frequently down-regulated in a p53-dependent manner in senescent cells [25]. IFI16 is a sensor of foreign or damaged cellular DNA and is capable of activating the inflammasome, but it is best studied as a sensor of viral DNA [59]. More on the role of p53 in the regulation of pyroptosis and other forms of non-apoptotic cell death pathways can be found in a recent review [60].

## 5. Genes of Bacteriostatic Proteins Activated by p53

Defensins are small, cysteine-rich cationic proteins expressed in both plants and animals where they are produced by epithelial cells and fight various infectious agents including bacteria, fungi, and viruses. The transcriptomic analysis found that A+N strongly up-regulates the expression of the *DEFB1* gene in A549 cells (Table 1). This gene encodes defensin beta 1. The expression fold change is “infinity” because the gene is not active in cells that grow under control conditions. Actinomycin D and nutlin-3a strongly synergize in the up-regulation of *DEFB1*, which is not up-regulated by actinomycin D or nutlin-3a acting alone. It is also up-regulated by A+N in the NCI-H460 cell line; however, the fold change is only slightly greater than threefold, probably because the gene is expressed in NCI-H460 cells grown under control conditions. The gene is neither up-regulated in the A375 melanoma cell line nor in the U-2 OS osteosarcoma cell line, which was frequently used by other investigators in the high-throughput search for p53-regulated genes, and probably for this reason its p53 expression score is low (three). There is also no evidence of p53 binding to or near the *DEFB1* gene [25]. *DEFB1* is also up-regulated by UVC radiation [61]. The role of p53 in this process was not investigated, but UVC was a well-known activator of p53 [62]. Thus, if this gene is regulated by p53, it occurs only in a narrow range of cell types or stress conditions. Interestingly, Ryan and Diamond [63] showed that the *DEFB1* promoter can be up-regulated by the IRF7 transcription factor. Our transcriptomic analysis showed that A + N up-regulates the *IRF7* gene nine times [5]. Therefore, it can be hypothesized that *DEFB1* is indirectly up-regulated by p53 via the IRF7 protein.

The possibility that p53 up-regulates this gene is intriguing because *DEFB1* encodes a very interesting antimicrobial peptide that exists in multiple forms, ranging from 36 to 47 amino acids, depending on where in the body they were isolated. The gene is constitutively expressed in various epithelia, including lung epithelium and the female reproductive tract. The molecule is the first line of defense against invading bacteria as well as fungi and viruses. According to some opinions, it is the most important antimicrobial peptide in epithelial tissues [64]. The gene is also expressed in monocytes, monocyte-derived macrophages, and monocyte-derived-dendritic cells. In monocyte-derived macrophages, its expression is strongly up-regulated by lipopolysaccharide and IFN-γ [65]. DEFB1 kills Gram-positive and Gram-negative bacteria, and *Mycobacterium tuberculosis*. It can kill bacterial cells directly by binding to negatively charged cytoplasmic membranes and disrupting their integrity (reviewed by [66]). It can also chemotactically attract dendritic cells and T lymphocytes and promote NET formation by neutrophils, which also kill bacteria. Furthermore, DEFB1 can even inhibit HIV-1 replication (reviewed by [67]). Mice deficient in the murine version of DEFB1 show delayed clearance of *Haemophilus influenzae* from the lung [68]. Interestingly, DEFB1 can also affect male fertility by influencing the quality of human spermatozoa. Deficiency of this protein is associated with the presence of leukocytes in the sperm (leukocytospermia, a sign of infection) and with reduced spermatozoa motility [69].

A very interesting group of genes regulated by p53 belongs to the LCE1 (late cornified envelope) family. These genes encode small cationic epidermal proteins with antimicrobial properties. Their regulation by p53 was first discovered by Deng et al. [51]. They are located in a cluster on chromosome 1 in a region called the epidermal differentiation complex and are constitutively expressed in the stratum corneum of the epidermis [70]. However, they (i.e., *LCE1A*, *LCE1B*, *LCE1C*, *LCE1E*, and *LCE1F*) can be up-regulated in lung cancer cell lines that overexpress p53 from the adenoviral vector, and in HCT116 colon cancer cell lines exposed to UV radiation [51]. Transcriptomic data from cells exposed to actinomycin D, nutlin-3a, or both compounds (A+N) mostly confirmed these previous findings, showing that in A549 cells the expression of this group of genes (*LCE1F*, *LCE1E*, *LCE1C*, and *LCE1B*) was undetectable in control conditions but was strongly induced following exposure to A+N [5]. They were also up-regulated by actinomycin D and nutlin-3a acting alone, but the degree of activation was not as high as during the combined treatment because there is a strong synergy between actinomycin D and nutlin-3a in their activation. The other genes in the cluster (*LCE1D* and *LCE1A*) were also up-regulated after treatment with A+N, but their expression was not prominent. A similar expression pattern is observed in control cells and during exposure to A+N in another lung cancer cell line (NCI-H460), in U-2 OS osteosarcoma cells, and in A375 melanoma cells (Table 1). Therefore, this gene cluster can be up-regulated by A+N in four cell lines derived from different histological origins. These genes have a moderate p53 expression score: *LCE1F*—17, *LCE1E*—18, *LCE1C*—20, and *LCE1B*—24. High-throughput analyses also show that promoters of some of these genes bind p53 [25]. Thus, there is strong evidence that p53 directly activates several genes of this cluster. To our knowledge, this is the only cluster of related genes known to be activated by p53.

There is only circumstantial evidence that the LCE1 subgroup of the LCE cluster has antimicrobial properties, but broad-spectrum antimicrobial activity has been directly shown in the LCE3 group [71]. LCE3 proteins interact with the CYSRT1 protein, which is expressed in skin and epithelial tissues and has antimicrobial activity against *Pseudomonas aeruginosa*. CYSRT1 also interacts with all examined members of the LCE1 group [72]. In general, the LCE1 proteins are poorly studied in contrast to the LCE3 group, which was intensely investigated because the deletion of two of its members is associated with the risk of psoriasis [73]. In conclusion, there is strong evidence that selected genes in the LCE1 group that encode late cornified envelope proteins are positively controlled by p53; however, direct microbial activity has only been demonstrated so far for members of the related group of LCE3 proteins [71]. The regulation of LCE1 genes by p53 suggests that they are important elements of the p53-regulated stress response system, and their functional role warrants further investigation.

Interestingly, the aforementioned CYSRT1 protein, also expressed in the stratum corneum and with confirmed antimicrobial activity, was found to be strongly activated by A+N in the lung cancer cell lines (Table 1), in the osteosarcoma cell line (U-2 OS), and in the melanoma cell line (A375) [5]. The p53 expression score of the *CYSRT1* gene is high (34) and both its promoter and enhancer are bound by p53, indicating that the gene is under the direct control of p53 [25]. Thus, the antibacterial activity of p53 may be partially mediated by the activation of the *CYSRT1* gene, whose protein is poorly studied—a PubMed search (17 March 2025) yields only two references.

Another group of proteins that can have antibacterial properties belongs to the WFDC (whey acidic protein four-disulfide core) family. These low-molecular-weight proteins are classically viewed as a family of proteins with roles as protease inhibitors and antimicrobial agents. Two of the best-studied members, SLPI and elafin, have antibacterial, antiviral, and anti-inflammatory properties. They are abundantly expressed in the human lungs, where they protect them from proteolytic attacks [74]. Our transcriptomic analysis revealed strong up-regulation of the *WFDC2* and *WFDC5* genes by A+N in A549 lung cancer cell lines and strong synergy between these two drugs in the up-regulation of these genes. Their activation by A+N or the anticancer drug camptothecin was prevented in p53-deficient cells [5]. Their p53 expression score is moderate (11 for *WFDC2*) or low (2 for *WFDC5*). P53 was not found to bind to its promoters or enhancers [25]. Thus, these genes are probably activated in a p53-dependent manner, but it occurs in a subset of cell types and may be indirect. Consistent with this conclusion, in another lung cancer cell line, NCI-H460, A+N only strongly up-regulates *WFDC5* from zero expression in control cells. In U-2 OS cells, *WFDC2* but not *WFDC5* is activated, whereas in the melanoma cell line (A375) neither of these genes is activated [5].

The functions of WFDC2 and WFDC5 are not studied as extensively as SLPI and elafin, which exert antimicrobial activity against a range of Gram-positive and Gram-negative bacteria, including *Pseudomonas aeruginosa* and *Staphylococcus aureus*. The biochemical mechanisms that explain the antimicrobial activity are a matter of speculation. According to one hypothesis, the cationic nature of WFDC proteins allows them to interact and disrupt negatively charged bacterial cell membranes [74]. The antibacterial properties of WFDC2 and WFDC5 are poorly explored. Serum levels of the WFDC2 (HE4) protein have been widely investigated as a potential biomarker of ovarian cancers [75]. In vitro, purified WFDC2 slightly inhibits the growth of *Staphylococcus aureus* and inhibits the secretory proteinases of *Bacillus subtilis* [76]. Previous animal studies demonstrated that WFDC2, expressed in milk, showed antibacterial activity against *Staphylococcus aureus*, *Salmonella enterica*, and *Pseudomonas aeruginosa* but not against *Enterococcus faecalis*. The authors speculated that this pattern of antibacterial activity protects the mother against mastitis caused by *S. aureus* or protects the gut of the young at a time when it is not immune competent: against pathogenic bacteria but not against commensal *E. faecalis* [77]. WFDC2 is highly expressed in pulmonary tissue, which may indicate a role for this WFDC protein in lung homeostasis and disease. Due to its similarity with SLPI and elafin, WFDC2 is proposed to play a role in the innate immune defense of the respiratory system (reviewed by [74]). Recent studies also hint at the antibacterial activity of WFDC2. It was found to preserve the integrity of tight junctions between colonic epithelial cells preventing invasion by commensal bacteria and mucosal inflammation [78]. In humans, several individuals with recessive *WFDC2* mutations were identified. These patients suffer from bronchiectasis, a pulmonary disorder defined by persistent pathological dilatation of the bronchi associated with chronic cough, sputum production, and recurrent respiratory infections. In addition to bronchiectasis, these individuals showed severe rhinosinusitis, nasal polyposis, and pulmonary *P. aeruginosa* infection [79]. This observation suggests that WFDC2 has some non-redundant function and that related WFDC proteins cannot substitute for its deficiency, which in humans is clearly associated with chronic lung infections. The link between the *WFDC2* gene with chronic rhinosinusitis with nasal polyps was also confirmed by other authors [80]. In conclusion, there is strong evidence that a part of the antibacterial activity of p53 involves up-regulation (direct or indirect) of the *WFDC2* gene; however, the exact mechanism of antimicrobial activity of its protein is not known. The function of the related protein, WFDC5, is also poorly studied—as of writing this (17 March 2025) the PubMed search yields only seven references. It is up-regulated in mice with lipopolysaccharide-induced epididymitis [81]. Moreover, it is a prognostic marker in melanoma—its high expression is associated with worse survival [82]. However, there is no direct evidence that this protein has antibacterial activity demonstrated for related proteins. Since the *WFDC5* gene apparently belongs to the p53 transcriptional program, its function deserves more scrutiny.

## 6. p53 Promotes the Detection and Destruction of Bacteria

Toll-like receptors (TLRs) are proteins that play a key role in innate immunity. They are usually (but not exclusively) expressed in sentinel cells, such as macrophages and dendritic cells, where they recognize molecular patterns characteristic of infectious agents (e.g., lipopolysaccharides typical for bacteria or long, double-stranded RNA characteristic for viruses). Some TLRs are localized on the cell surface, while others are in intracellular vesicles (endosomes). Ligand binding by TLR initiates a signaling cascade mediated by MYD88 and other proteins, which culminates in the activation of transcription factors of the NF-κB family and the secretion of pro-inflammatory cytokines. Some TLRs (e.g., TLR3) use a different signaling pathway, which includes the TRIF protein and the transcription factors IRF3 and IRF7 and culminates in the production of type I interferons (e.g., IFN-β). This is only a crude outline of these signaling systems, which are in fact very complex [83]. The p53 protein is known to activate the *TLR3* gene expression [52]. Transcriptomic data support this observation because actinomycin D and nutlin-3a acting alone and in combination up-regulate *TLR3* expression in the A549 cell line, and A+N up-regulates *TLR3* expression in melanoma (A375), osteosarcoma (U-2 OS), and another lung cancer (NCI-H460) cell line (Table 1) [5]. *TLR3* has a relatively high p53 expression score (26), and its promoter is bound by p53, which is strong evidence that this gene is directly activated by p53 [25]. Interestingly, *TLR3* is robustly up-regulated in A549 cells by infection with the respiratory syncytial virus [84], although it is not known whether p53 mediates this up-regulation. TLR3 is located in endosomes, where it recognizes double-stranded RNA derived from viruses. Therefore, it may appear that stimulation of *TLR3* by p53 does not modulate the cell response to bacteria because TLR3 recognizes predominantly viral infections. However, there is a connection between TLR3, p53, and the bacterium, namely *Moraxella catarrhalis*. It is one of the most frequently detected lower respiratory tract pathogens in patients with chronic obstructive pulmonary disease. *M. catarrhalis* induces a significant down-regulation of TLR3 in human bronchial epithelial cells, which is associated with decreased expression of p53. The down-regulation of TLR3 is probably caused by reduced p53 binding to the *TLR3* promoter. Consequently, *M. catarrhalis* decreased the TLR3-dependent secretion of IFN-β [85]. Thus, in this very interesting example, bacterial infection increases the risk of viral infections by reducing the p53-dependent expression of TLR3 and consequently the interferons. Furthermore, the fact that this bacterium reduces TLR3 expression suggests that this protein may somehow inhibit its replication. In line with this hypothesis, Campos et al. [86] demonstrated that TLR3 can recognize RNA purified from *Brucella abortus*, but it is dispensable for host control of infection. However, in an experiment carried out in another model, TLR3 was shown to be involved in cytokine production after infection with *Chlamydia*. Furthermore, *Chlamydia* replication in TLR3-deficient cells was more efficient than in wild-type oviduct epithelial cells [87]. TLR3 is also vital to macrophage response in the early stages of *Legionella pneumophila* infection [88]. Thus, although TLR3 is best studied as a detector of viral double-stranded RNA, it also plays a role in response to infections with various bacteria.

Transcriptomic analysis showed that the A+N combination strongly induces TLR2 expression in A549 and NCI-H460 lung cancer cells, however, it is not up-regulated in melanoma (A375) and osteosarcoma (U-2 OS) cell lines (Table 1). Actinomycin D and nutlin-3a synergize in *TLR2* up-regulation, indicating that p53 must be strongly activated to efficiently stimulate its expression [5]. The p53 expression score of *TLR2* is very low (two) [25]. Interestingly, data published by others indicate that it is up-regulated in the p53-null lung cancer cell line (NCI-H1299) with ectopically expressed p53 [25]. Thus, if p53 activates this gene it occurs under particular conditions and in a limited number of cell lines (possibly bronchial epithelium). The TLR2 receptor is expressed on the cell surface and, unlike many other TLRs that act as homodimers, it heterodimerizes with TLR1 or TLR6 to recognize various molecules present on the surface of the bacterial cell wall. It signals through the MYD88 protein, leading to activation of NF-κB and the expression of pro-inflammatory cytokines. In addition to bacterial molecules (mainly Gram-positive components of the bacterial wall), TLR2 binds endogenous cellular proteins, which serve as cell damage signals, e.g., heat shock proteins or HMGB1. Due to its role in defense against bacteria, it is an intensely studied molecule with more than 15,000 citations. Experiments with TLR2 knockout animals show that this receptor is crucial for defense against *Streptococcus pneumoniae* [89] and *Staphylococcus aureus* [90]. TLR2 knockout mice infected with *Mycobacterium tuberculosis* survive but produce fewer cytokines [91]. Despite reduced innate immunity, acquired immunity, as far as it has been studied, functions properly [92]. The functions of toll-like receptors are best studied in macrophages because they are the sentinel cells that recognize invading pathogens. However, TLRs, including TLR2, are also expressed in bronchial epithelial cells, which can recognize and mount a TLR-dependent response against invading bacteria. Interestingly, bronchial epithelial cells respond only to some TLR2 ligands [93]. On the other hand, human intestinal epithelial cells do not respond to TLR2 stimulation by its ligands secondary to deficient receptor expression [94]. This probably serves to prevent chronic pro-inflammatory cytokine secretion in response to commensal Gram-positive bacteria in the gut. Notably, a cell line commonly used to study p53 expression (HCT116) is derived from colon cancer and these cells may be generally resistant to TLR2 up-regulation. Overall, the evidence for *TLR2* regulation by p53 is preliminary, but existing data suggest that it may be cell-type specific, e.g., it may occur in cells derived from bronchial epithelia.

The *ACP5* gene codes for the secreted protein named acidic phosphatase 5. It is strongly up-regulated by the A+N combination in A549, NCI-H460, U-2 OS, and A375 cell lines (Table 1). Interestingly, in contrast to other cell lines, it is expressed in melanoma cells (A375) growing in normal conditions and is further up-regulated after exposure to A+N. The protein was detected by mass spectrometry in the secretome of A549 cells exposed to A+N, but not in cells grown under control conditions [5]. Considering that mass spectrometry primarily detects abundant proteins, its amount in the medium of treated cells is likely to be relatively high. The *ACP5* gene has a moderate p53 expression score (10), probably because it is not up-regulated by nutlin-3a [5], and it is frequently used in the high-throughput search for p53-regulated genes. It has no known p53 binding sites in the promoter or enhancer [25]. However, RT-PCR tests demonstrated that ACP5 up-regulation by A+N or camptothecin (anticancer drug) is prevented in p53-deficient cells [5]. Antimicrobial activity of ACP5 was first observed by Bune et al. [95], who prepared *Acp5* knockout mice and observed that they showed delayed clearance of *Staphylococcus aureus* after sublethal intraperitoneal inoculation, which was probably caused by impaired recruitment of macrophages to the site of infection. Similar conclusions were drawn using a different experimental model, in which Acp5 knockout mice (and their controls) received intratracheal administration of *P. aerugniosa*. The knockout animals had impaired clearance of airway infection and reduced recruitment of immune cells (i.e., neutrophils and inflammatory macrophages) [96]. In humans, ACP5 was found to be a candidate marker for immune reactions to leprosy [97]. Therefore, by activating *ACP5*, p53 can help to recruit macrophages and other immune cells to the site of infection.

Autophagy is a process by which selected intracellular content is first enclosed in membrane vesicles, which are subsequently fused with lysosomes containing various hydrolytic enzymes that digest the content of the vesicles. The process is initiated when the energetic content of cells, measured, for instance, as an ATP/AMP ratio, drops dangerously. This provides energy for the starving cell and enables its survival. Autophagy is also initiated to remove damaged organelles, which can be dangerous to cells. The best-studied example is mitophagy, which removes damaged or excess mitochondria. Finally, autophagy is used to remove intracellular bacteria and viruses, which is called xenophagy. It must be stressed here that bacterial xenophagy must be distinguished from efficient phagocytosis. When bacteria escape from the phagosomal compartment upon phagocytosis, they may be removed by xenophagy. In mammalian cells, cytoplasmic bacteria are rapidly recognized by various autophagy receptors including p62, NDP52, TAX1BP1, and OPTN (optineurin) [98]. The *OPTN* gene has a high p53 expression score (31), and its enhancer is bound by p53 [25]. Transcriptomic data show that it is up-regulated by A+N in the A549, NCI-H460, and A375 cell lines (Table 1). Thus, it is very likely that the optineurin gene is directly controlled by p53 in various cell types.

As with many other genes, important *OPTN* functions were discovered by the observation of knockout animals and by the epidemiological, molecular, and genetic analyses of human individuals. Crohn’s disease (CD) is a chronic inflammatory disorder that affects the gastrointestinal tract. In CD patients the clearance of bacteria from their tissues is defective, which is associated with inadequate neutrophil recruitment and is probably caused by impaired secretion of pro-inflammatory cytokines from macrophages at sites of bacterial invasion. To elucidate the molecular mechanism, the researchers performed a transcriptomic analysis of macrophages from individuals with CD. *OPTN* was identified as a gene with abnormally low expression in approximately 10% of CD patients. Other experiments demonstrated that OPTN promoted the secretion of pro-inflammatory cytokines (TNFα and IL-6) from macrophages after bacterial stimulation ([99] and references therein). It has been further shown that in OPTN-deficient cells, TNFα is mistrafficked to lysosomes, hence its lower secretion. These observations suggest that p53, by activating OPTN expression, indirectly promotes the recruitment of neutrophils to the sites of infection. Observations of *OPTN* knockout mice demonstrated that the animals were more susceptible to *Citrobacter* colitis and *E. coli* peritonitis, showed reduced levels of TNFα in serum, had decreased neutrophil recruitment to sites of acute inflammation, and had greater mortality [99]. These findings indicate that OPTN combats bacterial infections by promoting the secretion of pro-inflammatory cytokines. OPTN-deficient mice are more susceptible to *Salmonella* infection [100]. Experimental data also demonstrate that optineurin participates in the xenophagy of cytosolic *Salmonella enterica* [101]. It is noteworthy that OPTN is not only associated with infections but also with several other diseases such as rheumatoid arthritis, osteoporosis, and nephropathy. OPTN tends to be protective in most autophagy-associated diseases, although the molecular mechanism of OPTN regulation in these diseases is poorly understood. Thus, p53 through regulating OPTN may modulate the course of all these diseases. More details on the role of optineurin in autophagy and human disorders are presented in a recent review [102].

## 7. TNFRSF14, p53, and Bacteria

**Figure 4:**
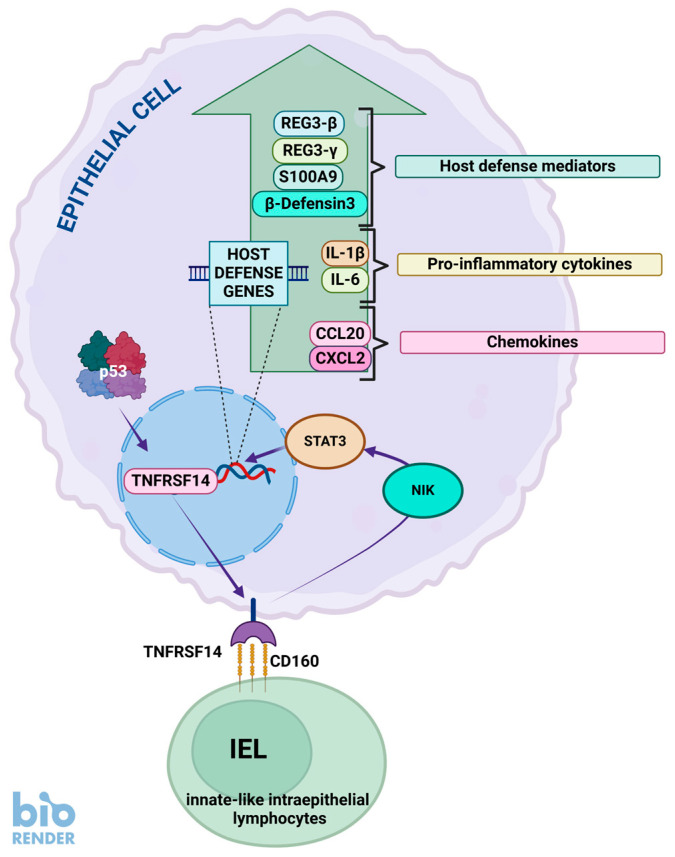
The antibacterial role of p53, which is mediated by up-regulation of the *TNFRSF14* gene. This gene codes the cell surface protein, which can either act as a ligand or as a receptor depending on the cellular context. In this example, TNFRSF14 protein acts as a receptor for CD160 protein expressed on innate-like intraepithelial lymphocytes. Stimulation of the receptor by CD160 protein triggers a cascade of events involving the activation of NIK kinase (NF-κB-inducing kinase), which phosphorylates and activates the STAT3 protein. This transcription factor induces a plethora of host defense genes, e.g., the gene for β-defensin 3, the genes for pro-inflammatory cytokines and chemokines.

The *TNFRSF14* gene codes for the TNF receptor superfamily member 14 (Figure 4). It mediates the entry of herpes simplex virus (HSV) into cells; hence its alternative name is HVEM (herpesvirus entry mediator). It functions in signal transduction pathways, signaling via the TRAF2-TRAF3 E3 ligase to regulate the survival, differentiation, and activation of immune cells. TNFRSF14 is bound by several proteins, such as BTLA, CD160, LIGHT, and lymphotoxin-α (LTα). The binding of LIGHT or LTα delivers a co-stimulatory signal, while the binding of BTLA and CD160 delivers a co-inhibitory signal [103]. However, it must be stressed that the TNFRSF14 protein can act as both a ligand and a receptor. For instance, it functions as a ligand for the BTLA and CD160 proteins, and as a receptor which activates NF-κB signaling in response to BTLA, CD160, LIGHT, and LTα (reviewed by [104]).

This gene is strongly up-regulated (145 times) by A+N treatment in A549 cells, as well as by actinomycin D and nutlin-3a acting alone. Up-regulation by A+N also occurs in U-2 OS, NCI-H460, and A375 cells, hence regulation of *TNFRSF14* by p53 is not limited to one cell line. This gene has a relatively high p53 expression score (34), so its activation by p53 in a large proportion of cells and stress conditions is well established [25]. The p53 binding sites were not detected in the vicinity of TNFRSF14, so it is not obvious if p53 regulates its expression directly; however, the high-throughput data published by others and our own experiments strongly suggest that p53 participates in its activation, e.g., up-regulation of this gene is strongly attenuated in p53-deficient cells [5].

TNFRSF14 has various functions in the immune system, augmenting or inhibiting its activity depending on the context. However, its role in antibacterial defense was clearly shown in experiments carried out by Shui et al. [105]. Epithelial TNFRSF14 plays a role in the innate mucosal defense against pathogenic bacteria. The authors generated knockout mice and observed their reaction to various pathogenic bacteria. During intestinal *Cintrobacter rodentium* infection, knockout mice showed attenuated activation of the STAT3 transcription factor, which is the main regulator of the immune system. Mice also showed higher bacterial burdens and increased mortality. This antibacterial protection was mediated by the CD160 protein expressed by innate-like intraepithelial lymphocytes acting as a ligand for epithelial TNFRSF14 (Figure 4). This protein provided a similar host defense during pulmonary *Streptococcus pneumoniae* infection. The expression of the activated STAT3 transcription factor in the lungs of animals infected with *S. pneumoniae* was significantly lower in *TNFRSF14* knockout mice compared to controls and so was the expression of the examined host defense genes, e.g., the IFN-γ gene. Expression of these genes can be induced by the TNFRSF14 agonist in lung epithelial cells [105]. Thus, p53, by promoting TNFRSF14, participates in the protection of mucosal epithelia (e.g., in the colon and lung) against bacterial infections. This may be particularly important during stress (e.g., viral infections), which on the one hand may damage the physical barrier against bacteria in epithelial layers but on the other hand may activate p53, which enhances protection by activating TNFRSF14. The important role of TNFRSF14 in antibacterial defense in humans is suggested by epidemiological and genetic observations showing that polymorphisms of this gene are associated with an increased risk of *Clostridium difficile* infection in ulcerative colitis patients [106].

From a biological point of view, it is intriguing why the p53 tumor suppressor activates TNFRSF14 because it acts as a ligand for the BTLA receptor expressed in T and B lymphocytes, inhibiting their activation. Therefore, the TNFRSF14-BTLA ligand–receptor pair is considered a plausible target for cancer immunotherapy [103]. On the other hand, overexpression of TNFRSF14 inhibits the proliferation of cancer cells, suggesting that this protein may be a tumor suppressor [107]. Furthermore, this gene is a tumor suppressor in a subset of lymphomas [108]. However, delving into the details of the role of TNFRSF14 in cancer biology is beyond the scope of this review. Details can be found in recent reviews [103,109]. In conclusion, p53, by activating TNFRSF14, may modulate the immune system, protect against bacterial infections, and inhibit cell proliferation.

## 8. p53 Promotes the Presentation of Bacterial Metabolites to Lymphocytes

The *MR1* (major histocompatibility complex class I-related) gene codes for the antigen-presenting molecule specialized in displaying microbial metabolites to T-cell receptors present on particular lymphocytes called innate-type mucosal-associated invariant T (MAIT)-cells. Thus, cells infected with bacteria and displaying a large number of MR1 molecules are the targets of MAIT lymphocytes. We have detected *MR1* as a plausible p53-regulated gene in our transcriptomic study [5]. The gene is activated not only by A+N but also by actinomycin D and nutlin-3a acting alone. Therefore, *MR1* is up-regulated by activated p53 in a “classical” fashion. We found that its promoter cloned in the reporter vector is activated by wild-type p53, but not by its inactive mutant. Activation of MR1 is significantly attenuated in cells deficient in p53. The gene is activated not only in A549 lung cancer cells but also in melanoma (A375), osteosarcoma (U-2 OS), and another lung carcinoma cell line (NCI-H460) [5]. This gene has a high p53 expression score (40), and its enhancer is bound by p53 [25]. Thus, there is very strong evidence that *MR1* is commonly and directly activated by p53. The functioning of MR1 has recently been described in a review article [110]. The foreign molecules displayed for recognition within the class I major histocompatibility complex (MHC) are protein fragments. The lymphocytes whose receptors perfectly match the foreign protein displayed on MHC-I are stimulated for proliferation (clonal expansion) and are the ones that subsequently fight the invading microorganism. In the case of MR1, the foreign molecule is not a protein but a bacterial metabolite. These metabolites bound to MR1 are displayed to relatively abundant types of T-lymphocytes specialized in the recognition of MR1 molecules (MR1-restricted T-cells, MR1T). 

The first-identified MR1-associated ligands were metabolites of the vitamin B2 (riboflavin) biosynthesis pathway. This vitamin is synthesized by yeast and bacteria, but not by mammals. Therefore, the appearance of these metabolites in mammalian cells is a sign of infection. A subset of MR1T cells are MAIT cells—they recognize cells with riboflavin metabolites displayed on MR1. MAIT cells are relatively abundant in blood (1–10% of all T-cells) and are involved in wound healing, immunity to bacterial infection, and microbiome control. The strong evolutionary conservation of the MR1 antigen presentation system and the MAIT cell transcriptional program suggest important and non-redundant roles for MAIT cells in the regulation of immunity (reviewed by [111]). Notably, there are also other types of MR1T cells which recognize MR1-associated antigens on tumor cells; however, the nature of these tumor antigens is not known. Thus, by up-regulating MR1 in cancer cells, p53 may promote their recognition by these atypical MR1T cells.

In contrast with classical MHC-I molecules (over 10,000 alleles), MR1 shows very little inter-individual variability (only 6 allele groups; [112]), which creates a huge opportunity for the application of MR1T cells in the therapy of bacterial infections and cancer. Of the six allelic variants of MR1, only one (R9H) creates an amino acid substitution, which can affect antigen binding within MR1. An individual who was homozygous for the R9H variant of MR1 had a primary immunodeficiency and was found to lack the MAIT cell subset [113]. This protein variant did not present one of the vitamin B2-related metabolites. The limited allelic variation of MR1 compared to HLA makes it an attractive target for potential cancer therapies. For example, MAIT cells can be expanded ex vivo, or induced pluripotent stem-cell-derived MAIT cells can be prepared and potentially used in universal adoptive therapy in various settings, including hard-to-treat viral or bacterial infections or cancer (reviewed by [114]).

The therapeutic potential of MR1T cells has fueled an extensive search for MR1 ligands beyond vitamin B2-related molecules. Our observations suggest that these therapeutic MR1T cells could potentially be combined with the agents that induce MR1 expression in cancer cells expressing wild-type p53 protein. MR1 can present self-antigens to MR1T cells [115], and a single MR1T cell can kill a wide range of cancer cell lines and primary cancer cells while remaining inert to healthy cells [116]. Thus, cancer cells that express the cancer antigen in the context of MR1 are vulnerable to destruction by matching T-cells. Therefore, according to these experiments, expression of MR1 with the tumor antigen can cause cancer destruction by T-cells. Interestingly, MR1-restricted T-cells that respond to cancer cells can be isolated from all donors tested [112].

On the other hand, some animal experiments show that tumor initiation, growth, and experimental lung metastasis were significantly reduced in *Mr1* knockout mice, compared to wild-type animals [117], suggesting that MR1 expression promotes cancer at least in mice. This apparent discrepancy may partly result from important differences between murine and human MAIT cells (reviewed by [111]). More details on the anticancer role of MAIT cells can be found in a recent review by Sugimoto et al. [114].

MAIT cells are found in the circulation and virtually all tissues, but they are particularly enriched in mucosal organs, especially in the lungs. As mentioned earlier, in humans MAIT cells represent up to 10% of T-cells in the blood, while in the lungs their frequency increases up to 15%, which is not surprising as the lungs are exposed to a plethora of microorganisms. MAIT cells can be considered as sentinels during bacterial infections of the lung. Some of the best-characterized examples of the reaction of MAIT cells to invading bacteria include *Francisella tularensis* and *Legionella longbeachae*. MAIT cells accumulate in the murine lung after intranasal infection with these bacteria, and optimal activation and expansion are MR1-dependent. *MR1* knockout mice infected with these bacteria exhibit increased bacterial burden compared to wild-type animals. However, here is the twist, protection against *Legionella* depends on IFN-γ production by MAIT cells, while during *Francisella* infection MAIT cells promote pulmonary production of GM-CSF cytokine, which drives the differentiation of monocytes into dendritic cells, the recruitment of T-cells to the lungs, and the control of pulmonary bacterial growth (reviewed by [111]). Thus, the molecular response of MAIT cells to infection is pathogen-specific. Considering that stress generated by invading bacteria can activate p53, it is very likely that p53 promotes bacterial clearance by increasing the expression of MR1 in the cells at the site of infection. It is also likely that the stress generated in the lungs by abiotic factors, e.g., genotoxic compounds, also promotes MR1 expression via p53 activation in preparation for plausible infection through damaged epithelium. However, these hypotheses require direct testing. Given the robust activation of the *MR1* gene by p53 and the high abundance of MAIT cells in stress-prone lungs, *MR1* activation by p53 appears to be an important antibacterial function of this tumor suppressor.

## 9. Concluding Remarks

Many bacterial species infecting mammalian tissues developed mechanisms to inactivate p53, either by upregulating MDM2 activity, which results in p53 degradation, or by destabilizing p53 mRNA, resulting in reduced p53 synthesis. The fact that bacteria target p53 indicates that this tumor suppressor somehow impedes their growth. The studies on bacterial infections of p53 knockout mice suggest that p53 is a generally anti-inflammatory protein and infections are more harmful to p53-deficient cells compared to their wild-type controls. The exact mechanisms of the antibacterial activity of p53 are only starting to emerge. Activated p53 can alter the metabolic environment of cells, reducing the availability of nutrients that bacteria require for growth. P53 can also induce apoptosis, which kills the hosts of intracellular bacteria. However, the identification of many p53 target genes by high-throughput methods suggests that the cells have more p53-activated antibacterial weapons in their storage. For instance, cells can produce bactericidal proteins, the components of bacterial-sensing inflammasomes, or the factors that attract bactericidal cells like macrophages or neutrophils. Probably, there is no single most important antibacterial gene activated by p53, rather, there are many p53-activated genes contributing to the fight against bacteria, each in its own way. There are numerous p53-activated genes with completely unknown, but plausibly antibacterial, functions which were overlooked in experiments on animals in sterile laboratory conditions. Definitely, this area of research deserves more attention, especially in light of the appearance of antibiotic-resistant bacterial strains.

## Figures and Tables

**Table 1:** The genes coding for proteins with established or potential antibacterial functions and the clues for their regulation by p53. The p53-regulated genes identified in individual studies show signs of direct regulation by p53, e.g., their cloned promoters contain sequences matching the consensus sequence of a p53 response element and can be activated by p53 in luciferase reporter assays. If a gene was identified as a potential p53 target only in high-throughput studies, the surrogate marker of direct regulation is the binding of p53 to a gene promoter or enhancer detected by sequencing of immunoprecipitated DNA (ChIP-Seq). However, the lack of p53 binding in ChIP-Seq analysis does not preclude regulation by p53, as explained in the text.

Gene Name	p53 Expression Score (Max. 57) *	Activated by Actinomycin D and Nutlin-3a in Cell Lines ^#^	p53 Bound Promoter or Enhancer *	Regulation by p53 Detected in Individual Study	Antibacterial Function
*NLRP1*	33	A549, NCI-H460, U-2 OS,	yes	Krześniak et al. [47]	Pattern recognition receptor (PRR) of inflammasome able to detect bacteria.
*NLRP3*	6	U2-OS	no	Gong et al. [46]	PRR of inflammasome able to detect bacteria.
*CASP1*	15	A549, NCI-H460, U-2 OS, A375	yes	Gupta et al. [48], Schlereth et al. [49]	Common element of classic inflammasomes, activates cytokines and induces pyroptosis, activated by PRR recognizing either bacteria or viruses.
*GSDME*	31	NCI-H460, A375	no	Masuda et al. [50]	Pore-forming protein in plasma membrane triggering pyroptosis.
*DEFB1*	3	A549, NCI-H460,	no	no	Extracellular bactericidal activity and antimicrobial defense of epithelia.
*LCE1B*	24	A549, NCI-H460, U-2 OS, A375	yes	Deng et al. [51]	Constitutively expressed in epidermis and antimicrobial activity inferred from the function of related proteins from LCE3 group.
*LCE1E*	18	A549, NCI-H460, U-2 OS, A375	yes	Deng et al. [51]	Constitutively expressed in epidermis and antimicrobial activity inferred from the function of related proteins from LCE3 group.
*LCE1F*	17	A549, NCI-H460, U-2 OS, A375	no	Deng et al. [51]	Constitutively expressed in epidermis and antimicrobial activity inferred from the function of related proteins from LCE3 group.
*CYSRT1*	34	A549, NCI-H460, U-2 OS, A375	yes	no	Constitutively expressed in stratum corneum of epidermis, where it may contribute to antimicrobial host defenses.
*WFDC2*	11	A549, U-2 OS	no	no	Extracellular protease inhibitor with antimicrobial activity of poorly studied mechanism.
*WFDC5*	2	A549, NCI-H460	no	no	Plausible antimicrobial activity similar to WFDC2.
*TLR2*	2	A549, NCI-H460	no	no	Pattern recognition receptor, expressed on cell surface, heterodimerizes with TLR1 and TLR6, recognizes bacterial molecules, activates NF-κB transcription factors, and promotes the expression of pro-inflammatory cytokines.
*TLR3*	26	A549, NCI-H460, U-2 OS, A375	yes	Taura et al. [52]	Pattern recognition receptor, detects double-stranded RNA derived from viruses, and probably from bacteria, and signals through IRF3 and IRF7 transcription factors activating expression of type I interferons.
*ACP5*	10	A549, NCI-H460, U-2 OS, A375	no	no	May help to recruit immune cells (macrophages, neutrophils) to the site of infection.
*OPTN*	31	A549, NCI-H460, A375	yes	no	May participate in autophagy (xenophagy) of bacteria; indirectly helps to recruit neutrophils to the site of infection.
*TNFRSF14*	34	A549, NCI-H460, U-2 OS, A375	no	no	Member of the tumor necrosis factor receptor superfamily; may also function as a ligand. Complicated role in the regulation of immunity and cell growth; signaling receptor on epithelial cells for CD160 ligand expressed on intraepithelial lymphocytes, triggering the production of antimicrobial proteins and pro-inflammatory cytokines.
*MR1*	40	A549, NCI-H460, U-2 OS, A375	yes	no	Major histocompatibility complex class I-Related gene protein; the antigen-presenting molecule specialized in displaying microbial metabolites to T-cell receptors present on specialized lymphocytes called innate-type mucosal-associated invariant T (MAIT)-cells.

* According to Fischer et al. [25], **^#^** according to Łasut-Szyszka et al. [5].
